# Biological Role of Nutrients, Food and Dietary Patterns in the Prevention and Clinical Management of Major Depressive Disorder

**DOI:** 10.3390/nu14153099

**Published:** 2022-07-28

**Authors:** Miguel A. Ortega, Óscar Fraile-Martínez, Cielo García-Montero, Miguel Angel Alvarez-Mon, Guillermo Lahera, Jorge Monserrat, Maria Llavero-Valero, Luis Gutiérrez-Rojas, Rosa Molina, Roberto Rodríguez-Jimenez, Javier Quintero, Melchor Alvarez De Mon

**Affiliations:** 1 Department of Medicine and Medical Specialities, University of Alcala, 28801 Alcalá de Henares, Spain; oscarfra.7@hotmail.com (Ó.F.-M.); cielo.gmontero@gmail.com (C.G.-M.); maalvarezdemon@icloud.com (M.A.A.-M.); guillermo.lahera@gmail.com (G.L.); jorge.monserrat@uah.es (J.M.); mademons@gmail.com (M.A.D.M.); 2 Ramón y Cajal Institute of Sanitary Research (IRYCIS), 28034 Madrid, Spain; 3 Cancer Registry and Pathology Department, Hospital Universitario Principe de Asturias, 28805 Alcalá de Henares, Spain; 4 Department of Psychiatry and Mental Health, Hospital Universitario Infanta Leonor, 28031 Madrid, Spain; mariallaverovalero@gmail.com (M.L.-V.); fjquinterog@yahoo.es (J.Q.); 5 Psychiatry Service, Center for Biomedical Research in the Mental Health Network, University Hospital Príncipe de Asturias, 28806 Alcalá de Henares, Spain; 6 Department of Psychiatry and CTS-549 Research Group, Institute of Neuroscience, University of Granada, 18071 Granada, Spain; gutierrezrojasl@hotmail.com; 7 Psychiatry Service, San Cecilio University Hospital, 18016 Granada, Spain; 8 Department of Psychiatry and Mental, Health San Carlos University Hospital (HCSC), 28034 Madrid, Spain; rosamolina18@hotmail.com; 9 Research Biomedical Fundation of HCSC Hospital, 28034 Madrid, Spain; 10 Department of Psychology, Comillas University, Cantoblanco, 28015 Madrid, Spain; 11 Department of Legal Medicine, Psychiatry, and Pathology, Complutense University (UCM), 28040 Madrid, Spain; roberto.rodriguez.jimenez@gmail.com; 12 Institute for Health Research 12 de Octubre Hospital, (imas12)/CIBERSAM-ISCIII (Biomedical Research Networking Centre in Mental Health), 28041 Madrid, Spain; 13 Immune System Diseases-Rheumatology, Oncology Service an Internal Medicine, University Hospital Príncipe de Asturias, (CIBEREHD), 28806 Alcalá de Henares, Spain

**Keywords:** major depressive disorder (MDD), dietary interventions, omega 3 polyunsaturated fatty acids, polyphenols

## Abstract

Major Depressive Disorder (MDD) is a growing disabling condition affecting around 280 million people worldwide. This complex entity is the result of the interplay between biological, psychological, and sociocultural factors, and compelling evidence suggests that MDD can be considered a disease that occurs as a consequence of an evolutionary mismatch and unhealthy lifestyle habits. In this context, diet is one of the core pillars of health, influencing multiple biological processes in the brain and the entire body. It seems that there is a bidirectional relationship between MDD and malnutrition, and depressed individuals often lack certain critical nutrients along with an aberrant dietary pattern. Thus, dietary interventions are one of the most promising tools to explore in the field of MDD, as there are a specific group of nutrients (i.e., omega 3, vitamins, polyphenols, and caffeine), foods (fish, nuts, seeds fruits, vegetables, coffee/tea, and fermented products) or dietary supplements (such as S-adenosylmethionine, acetyl carnitine, creatine, amino acids, etc.), which are being currently studied. Likewise, the entire nutritional context and the dietary pattern seem to be another potential area of study, and some strategies such as the Mediterranean diet have demonstrated some relevant benefits in patients with MDD; although, further efforts are still needed. In the present work, we will explore the state-of-the-art diet in the prevention and clinical support of MDD, focusing on the biological properties of its main nutrients, foods, and dietary patterns and their possible implications for these patients.

## 1. Major Depressive Disorder and Diet: What Is the Relationship?

### 1.1. A General Perspective of Major Depressive Disorder

Major Depressive Disorder (MDD) is a leading disabling condition highly representative in our society, affecting nearly 280 million people worldwide [1]. Currently, MDD is placed as the third cause of disability globally, just after headache and lower back pain; however, the World Health Organization (WHO) projects that by 2030, MDD will become the first cause of disability worldwide [2,3]. Nowadays, the growing prevalence and annual incidence affected by pandemic times of the coronavirus disease 2019 (COVID-19) has made us reconsider the importance of mental health. In fact, according to current data, there has been a rise of 53.2 million MDD cases globally, representing an increase of 27.6% during the course of this pandemic [4]. This condition affects people from all age ranges, sex, and cultures; although, being aged between 45 and 59, of the female sex, and from a high-income country are associated with a higher risk of suffering from MDD. In fact, prevalence is double in women than in men [5,6]. In addition, it seems that the distribution of MDD can be different across regions. Thus, the lifetime prevalence of MDD can vary between 2 and 21% with the highest rates in some European countries and the lowest in certain Asian countries [7].

The precise causes of this complex malady are not fully understood. It seems that MDD is the result of an interplay between genetic and environmental factors. The estimated role of genetics is about 37%; although, a single gene has not been identified as a causative factor of MDD [8]. Instead, it seems that the inheritance of MDD is polygenic, the exposure to different environmental factors in certain critical periods being equally required [9]. In this sense, early life stress (i.e., child abuse) is one of the most important environmental factors related to the development of MDD, leading to determinant changes in the brain structure and function [10]. Similarly, chronic stress and frequent episodes of acute stress are also related to the onset of MDD [11]. The presence of physical or mental comorbidities together with multiple social factors can be equally prominent contributors to those suffering from MDD. In this sense, ethnic minorities, marital status (separated/divorced), poor education and healthcare access/quality, neighborhood, built environment, intimate partner violence, and lower socioeconomic resources are the most important social determinants related to the onset of MDD [7,12,13].

From a medical perspective, MDD can be considered a quite heterogeneous entity. Following the DSM-5 (Diagnostic and Statistical Manual of Mental Disorders, 5th Edition) criteria, MDD is clinically diagnosed by the presence of at least one of the two main criteria—depressed mood or anhedonia (the inability to feel pleasure or loss of interest) and ≥ 4 secondary symptoms during a minimum period of 2 weeks [14]. Collectively, the secondary symptoms of MDD can be divided into somatic or non-somatic items. The former include sleep disturbances, appetite changes, poor concentration, fatigue, and psychomotor agitation/retardation, whereas non-somatic symptoms are related to psychobehavioral alterations such as feelings of worthless, and suicide thoughts [15] The severity of MDD is generally assessed by the use of the Hamilton Depression Rating Scale (HAM-D), based on the use of specific punctuation marks for different clinical parameters [16]. Thus, a rating scale between 0 and 7 is defined as no depression; 8 and 16 mild depression; 17 and 23 moderate depression; and ≥24 severe depression [17,18]. The clinical management of these patients can be different according to the severity. Hence, for patients with mild depression, physical activity, or non-medical treatments may be encouraged. However, for patients with moderate or severe depression, the use of antidepressants, alone or in combination with psychotherapy and psychoeducation, is generally the most frequent approach [19].

On the other hand, notwithstanding an important percentage of patients responding to the therapy received, approximately 30% of patients suffer from resistance, which is known as treatment-resistant depression, representing an important medical and socioeconomic challenge [20]. In addition, patients with severe depression often require more intervention visits, more months of antidepressant treatment, and more antidepressant trials, but they tend to present worse clinical outcomes [21]. In this context, the socioeconomic burden of MDD is notably high, and not only are direct costs derived from their clinical management, but also indirect costs related to their lack of productivity or work absence must be considered. In the United States alone, the economic burden of MDD was USD 326.2 billion between 2010 and 2018, with a substantial rise in the indirect costs during this period [22].

Therefore, MDD is a challenging and growing public health concern that has a devastating impact on the individuals who suffer from this condition and the entire society. It is imperative to find novel approaches that facilitate the prevention and clinical management of this neuropsychiatric disorder, and nutritional intervention may represent a promising key to achieve these objectives.

### 1.2. Nutritional Status of the Patient with MDD

Malnutrition in form of undernutrition, overnutrition, or an imbalance of specific nutrients is a global community concern [23]. In general, children suffer more frequently from undernutrition, whereas adults tend to present overnutrition, especially in low- or middle-income countries and the poor population [24]. Likewise, malnutrition is remarkably higher in elder subjects, particularly with specific nutrient deficiencies [25]. It seems that there is a bidirectional link between malnutrition and MDD. On the one hand, malnutrition can drive several biological changes that can lead to the onset and progression of MDD. On the other hand, MDD can drive to eating disorders and lifestyle behaviors that may promote malnutrition [26]. However, there is an important association between malnutrition and MDD that can be observed in these patients.

Nutritional questionnaires such as the Mini Nutritional Assessment (MNA) have been useful to understand the connection between both entities. MNA represents a useful tool for monitoring patients of any age who are at risk of malnutrition. In this sense, rating as “malnourished” or “at risk of malnutrition” is associated with a higher risk of MDD, especially in elder individuals [27,28]. Moreover, some studies have found that patients with MDD are more likely to be at risk of undernutrition rather than overnutrition [29].

Simultaneously, the introduction of anthropometric variables has also aided in further insights being gained into the relationship between malnutrition and MDD. For instance, higher median levels of body weight, waist circumference, hip circumference, and waist-to-hip ratio were found in patients with MDD [30]. In this line, the waist-to-hip ratio appears to be inversely related to suicidality and severity of depression in women with postnatal depression, denoting the relevance of anthropometric measures in the clinical management of MDD [31].

When combining biochemical plus anthropometric variables, some studies have found that women with MDD have higher triglyceride, aspartate aminotransferase (AST), blood urea nitrogen (BUN), and creatinine levels and lower high-density lipoprotein cholesterol (HDL-C), hematocrit, and red blood cell counts; while depressed men were more likely to have higher triglyceride levels, lower hematocrit, and BUN and, in general, they present with less height and weight than their control group [32]. Self-perception can also be different between women and men. Women are more prone to perceive their body as larger than it really is, whereas, in men, the perception is just the opposite, idealizing a larger body [33].

Regarding the lack or excessive intake of specific nutrients, it is common that patients with MDD have an aberrant distribution of macronutrients (carbohydrates, proteins, and fats) and a deficiency of critical micronutrients (vitamins and minerals). In this context, prior works have associated high consumption of carbohydrates and low intake of protein with MDD [34]. Refined carbohydrates are one of the most deleterious nutrients included in westernized diets, being associated with multiple disease conditions [35]. Likewise, a direct relationship between trans unsaturated fatty acids and depression risk has been demonstrated [36], and a high dietary intake of saturated fats is sufficient to promote depressive-like behaviors in animal models [37]. Conversely, low high-quality fat intake, such as omega 3 polyunsaturated fatty acids (ꞷ-3 PUFA), has been associated with a higher risk of MDD [38]. Thus, patients with MDD often present with an insufficient protein and high-quality fat intake and with overconsumption of refined carbohydrates and poor-quality fats. In this situation, deficiencies in multiple vitamins and minerals are importantly linked to MDD. Particularly, a low intake of vitamin D and from the B complex—prominently thiamine (B1), niacin (B3), pyridoxin (B6), folate (B9), and cyanocobalamin (B12)—are observed in patients with MDD [39,40] Regarding minerals, low calcium, magnesium, iron, and zinc consumption has been associated with MDD [41], whereas low potassium, phosphorus, and copper intake can also be related to depressive symptoms for women, but not for men [42].

Collectively, there is compelling evidence that patients with MDD are commonly malnourished, or at least at risk of malnutrition. Thus, as it will be subsequently discussed, patients with MDD can benefit from addressing their nutritional status, as these dietary changes may favorably influence the intricate biology of depression.

### 1.3. Biology of Depression: Is There a Role for Diet?

The numerous difficulties related to the clinical management of MDD may be a consequence of the complex pathophysiological signature of this neuropsychiatric condition. Traditionally, the most widely known biological mechanism implicated in the pathophysiology of MDD has been aberrant neurotransmitter functioning and, particularly, decreased monoamine levels. Monoamines are represented by serotonin, dopamine, and norepinephrine, acting in critical brain regions and mediating several perceptions and behaviors in the brain [43]. However, currently, it is widely accepted that monoamines only represent a single mechanism of a really intricate picture [44]. Apart from monoamines, abnormal functioning of other neurotransmitters, such as gamma-aminobutyric acid (GABA), glutamate, and acetylcholine, has been reported in patients with MDD [45]. The available literature also considers the relevance of altered neuropeptides in the pathophysiology of MDD, including galanin (GAL), cholecystokinin (CCK), neuropeptide Y (NPY), oxytocin (OXT), vasopressin (VP), neuropeptide S (NPS), and melanin-concentrating hormone (MCH) [46]. Likewise, alterations in the neuropeptide melatonin and circadian disruption are similarly reported in patients with MDD [47], collectively supporting that there are many neurotransmitters and neuropeptides that are not working properly in the brain of depressed patients.

Furthermore, impaired neurogenesis and neuroplasticity have also been observed in the brain of depressed patients, having been proposed as a major pathophysiological signature of MDD [48]. For instance, alterations in neurotrophic factors such as brain-derived neurotrophic factor (BDNF) or glial-derived neurotrophic factor (GDNF) are critically involved in these changes, affecting several cellular processes [49,50]. The aberrant neurogenesis and neuroplasticity are closely linked to the effects of psychological stress in the patient with MDD. In this sense, it is widely accepted that this condition is associated with severe dysfunction of the hypothalamus–pituitary–adrenal (HPA) axis, characterized by altered levels of cortisol and their regulators, cortisol release factor (CRF) and the adrenocorticotropin hormone (ACTH), entailing detrimental consequences for the brain and the entire organism [51].

Moreover, consistent damage to neuronal and glial cells has been described in the brain of patients with MDD, with an enhanced mitochondrial dysfunction, apoptosis, oxidative stress, and excitotoxicity [52,53] All these changes lead to structural, functional, and connectivity abnormalities in specific regions of the central nervous system (CNS), such as the hippocampus, amygdala, dorsomedial thalamus, dorsal and medial prefrontal cortex, the dorsal and ventral anterior cingulate cortex, the orbital frontal cortex, the insula, and the striatum and raphe nucleus [54], which ultimately are responsible for the behavioral and mood dysfunctions related to MDD.

Not only local, but also extra-brain affections are observed in the patient with MDD. The immune system is a pivotal player in the pathophysiology of this condition and an aberrant inflammatory response can be observed either in the brain (neuroinflammation), or at systemic levels [55,56,57,58]. Metabolic and endocrine alterations (insulin resistance, type 2 diabetes, obesity, etc.) are closely related to immune dysfunction and can influence at the onset and development of MDD [59]. Indeed, an exacerbated inflammatory response frequently co-occurs with endocrine, neural, and psychiatric alterations such as in MDD, which is studied in the field of psychoneuroimmunoendocrinology, explaining the biological link between MDD and systemic diseases [60]. Moreover, gut dysbiosis and an altered microbiota–gut–brain (MGB) axis are tightly linked with inflammation and the above-mentioned mechanisms, also representing a potential etiopathogenic mechanism of MDD [61,62]. Thereby, the pathophysiology of MDD is undoubtedly complex, explaining the clinical and translational difficulties of this condition.

**Figure 1:**
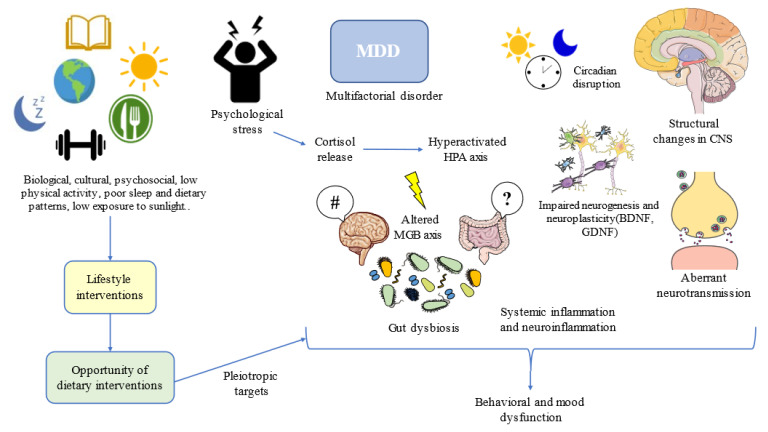
Lifestyle interventions such as dietary interventions suppose an opportunity for addressing multiple targets in the complex network of depression. MDD: Major Depressive Disorder; HPA: hypothalamic–pituitary–adrenal; CNS: central nervous system; MGB: microbiota–gut–brain; BDNF: brain-derived neurotrophic factor; GDNF: glial-derived neurotrophic factor. #/?: Aberrant gut brain crosstalk due to an impaired MGB axis.

Diet has pleiotropic effects in the organism. Depending on the content of specific nutrients and foods, as well as the whole dietary context, they may promote health or facilitate the onset and progression of multiple pathological conditions [35]. In the field of MDD, an umbrella review using 28 meta-analyses has defined the potential of nutrients, foods, beverages, and dietary patterns in the amelioration or progression of MDD; although, the quality of evidence is still moderate or low [63]. This means that further studies are warranted before drawing any definitive conclusion about the potential of diet and MDD. Indeed, some authors have claimed that narrative reviews tend to overestimate the impact of diet as preventive or as adjunctive support in this field [64], and we agree that is critical to be objective and put in context the promising but still inconclusive role of diet in MDD. What the evidence seems to indicate is that dietary interventions can aid in the alleviation of depressive symptoms, but most of these results have been obtained in patients without a clinical diagnosis of depression [65], so once again it should be understood that MDD is a complex and multifactorial disorder in which plenty biological, cultural, and psychosocial factors are interacting, being a huge challenge for healthcare professionals (Figure 1).

One of the most plausible explanations for the high prevalence of MDD and its relationship with diet can be perceived from an evolutionary context. Following the words of Theodosius Dobzhansky, “nothing in the biological aspects of medicine makes sense except in the light of evolution”. Evolutionary-based approaches have brought great advances in the understanding and clinical management of MDD [66]. MDD can be considered a disease that emerged as the result of low physical activity levels, sleep and circadian disturbances, low exposure to sunlight, social isolation, poor contact with nature, and, of course, due to poor dietary patterns [67]. Thus, lifestyle interventions following this evolutionary background have led to notable improvements in physical and mental health, while aiding to prevent the onset of mood disorders [68]. Moreover, addressing the entire picture from an evolutionary perspective, in combination with antidepressants and/or psychosocial support, along with additional medical therapy, if needed, can bring maximum benefits to patients with MDD (Figure 1).

In this context, the diet has the potential to modulate various pathophysiological mechanisms of MDD, including: (1) Amelioration of immune dysfunction and inflammation; (2) Favorable modulatory effects on gut microbiota and their derived products; (3) Limiting the impact of oxidative stress; (4) Enhancing mitochondrial function and displaying cytoprotective effects; (5) Boosting neurogenesis; (6) Epigenetic reprogramming; (7) Targeting HPA dysfunction; and (8) Influencing neurotransmitter and neuropeptides biosynthesis/degradation [69]. Additionally, diet has the potential to revert or promote specific biological mechanisms implicated in the pathophysiology of MDD in women, interacting with reproductive hormones throughout the menstrual cycle and playing a critical role during different critical stages such as pregnancy or menopause [70].

Hence, once the nutritional status of depressed patients, their background, and the actions of diet in the biology of MDD have been explored, the main nutrients, foods, and dietary habits studied in this field will be subsequently discussed.

## 2. Translational Opportunities: Clinical Management of MDD through Diet

### 2.1. Foods and Nutrients of Interest

Many efforts have been placed to find potential foods and dietary supplements with antidepressant effects as well as to prevent the onset of depression. These foods contain a wide variety of nutrients that act synergically to exert their benefits. Many times, these effects are different than those played by the nutrient alone. This concept is defined under the term food matrix, having numerous consequences for the global effect of foods [71]. Dietary supplements consist of an isolated nutrient with some interesting properties and both foods and supplements with potential preventive and supportive antidepressant activity can be defined as nutraceuticals, a growing area of research in the field of MDD [72]. In the field and clinical management of MDD, it is equally important to limit or avoid the consumption of ultra-processed foods, sugar-sweetened drinks, alcohol, as well as red and processed meats consumption, as they increase the risk of suffering from MDD [63]. In this part, we will focus on different food sources and supplements with potential preventive and antidepressant effects.

#### 2.1.1. Fish, Seeds, and Nuts

Compelling evidence is starting to support the benefits of fish intake in the management and prevention of MDD. Indeed, various meta-analyses show that moderate fish consumption was associated with a reduced risk of depression, especially in women ([73,74]. Furthermore, long-term intake of fish was also protective for elder individuals aged > 65 years, thereby supporting the benefits of fish consumption on mental health [75]. Due to their many bioactive components, fish intake is recommended as a key pillar of a healthy diet at least twice per week [76], or 350 g weekly including 200 g, which may be from oily fishes [77]. However, optimizing the most suitable recommendation of fish following preferences and costs is a major objective of current studies [78].

Fish and especially oily fishes as well as fish oil are rich in ω-3 PUFA, particularly docosahexaenoic acid (DHA; 22:6 ω-3) and eicosapentaenoic acid (EPA; 20:5 ω-3), proving multiple antidepressant effects [79]. Despite DHA and EPA sharing multiple biological actions, there are some disparities in their molecular activities explaining their differential role as antidepressants [80]. A meta-analysis of randomized control trials claimed that EPA, but not DHA, was responsible for the most antidepressant effects of ꞷ-3 PUFA [81]. Conversely, in a 5-year follow-up, it was demonstrated that the protective role against depression of high fish intake (≥2 times/week) was strongly influenced by the DHA content; although, other components rather than DHA may also contribute to the antidepressant effects of fish [82,83]. However, ꞷ-3 PUFA are well-known anti-inflammatory mediators, and this effect is importantly achieved by the alteration in epigenetic markers such as DNA methylation. [84]. In a similar manner, compelling evidence supports the benefits of ꞷ-3 PUFA due to its prebiotic effect, critically modulating the composition of the gut microbiota [85]. Other mechanisms involved in their antidepressant action include an increased membrane fluidity, leading to an increase in serotonin transport by endothelial cells, an enhanced dopamine concentration and dopamine 2 receptor in the frontal cortex, and direct interactions with other neuronal receptors and second messengers, exerting pleiotropic effects [86]. Other dietary sources of ꞷ-3 PUFA include seeds and nuts; although, they are frequently found in the form of α-linolenic acid (ALA; 18:3 ω-3), a precursor of EPA and DHA [87]. Because of that, some preclinical and observational studies have demonstrated some evidence about the use of seeds and nuts (especially walnuts) in the prevention and amelioration of depressive symptoms [88,89,90]. Specifically, 30 g/day of mixed nuts (15 g walnuts, 7.5 g hazelnuts, and 7.5 g almonds) comprised in a healthy dietary pattern appears to be an important recommendation to prevent MDD [91] Thus, ꞷ-3 PUFA consumption or supplementation has provided cumulative evidence of antidepressant and preventive effects in MDD; although, further studies are still required.

ꞷ-3 PUFA can present synergic effects with vitamin D in the brain, which is also abundant in seafood and oily fishes, helping to explain the antidepressant role of these foods [92]. Vitamin D is equally found in eggs and other animal products, mushrooms, or certain fortified products [93]. Despite its relevance to health, vitamin D intake is generally low worldwide, and supplementation with vitamin D is common or at least required in many population groups [94]. Specifically, low serum vitamin D levels appear to be inversely related to clinical depression [95]. In this context, notwithstanding some studies have found some favorable antidepressant benefits from vitamin D supplementation, these results are inconsistent with other works that did not report any protective or antidepressant effects of vitamin D [96,97]. The underlying biological mechanisms that help to explain the potential antidepressant role of vitamin D are through: (1) Immune and gut microbiota modulation; (2) Serotonin synthesis; (3) Circadian clock regulation; and (4) Augmented BDNF production [98,99,100]. In addition, sunlight exposure can aid in vitamin D production (but does not replace foods or supplementation with this nutraceutical), exerting synergic antidepressant actions proven in prior studies [101].

Apart from their high content in ω-3 PUFA and vitamin D, fish, seafood, seeds, and nuts are rich in high-quality proteins. A recent study has observed that a diet with high protein contents can interact with the rs7041 polymorphism of the vitamin D-binding protein (VDBP) and decrease the risk of severe depression [102]. Likewise, seafood, seeds, and nuts are also major sources of dietary zinc, along with other high protein foods such as legumes, poultry, and meat, aiding to prevent the onset of MDD [103]. Finally, a study conducted by Cediel Ulloa et al. [104] demonstrated that high prenatal exposure to methylmercury due to high fish consumption was associated with differential DNA methylation at seven years of age at specific CpG sites, with adverse consequences in neurodevelopment and mental health. During pregnancy, it should be important to limit the consumption of fish with high levels of mercury. Further studies are needed to unravel the effect of fish and its different components in the etiopathogenesis of MDD.

#### 2.1.2. Fruits, Vegetables, and Berries

Fruits and vegetables are also a group of foods widely explored in the prevention and therapeutic support of many diseases such as MDD. Current dietary guidelines recommend that these groups of foods may constitute about one-half of the plate at each meal and a total mean intake of 800 g per day [105]. However, there are some subgroups of fruits and vegetables more interesting for cognitive working and mental health, as is the case of berries, citrus, and green leafy vegetables [106]. Meta-regression and analytic data show that a 100 g increased intake either of fruit or vegetables was associated with a 3% reduced risk of depression [107]. Conversely, patients with MDD often eat fewer fruits, vegetables, and legumes than healthy subjects, while consuming more sweets and pastries [108]. The benefits of this group of foods can be attributed to three main components: (a) dietary fiber; (b) vitamins; and (c) polyphenols.

The first component, dietary fiber seems to exert critical actions in the gut microbiota, leading to the production of short-chain fatty acids (SCFAs) and lowering inflammation by modifying both the pH and the permeability of the gut. Importantly, these changes may be associated with a reduction in depressive symptoms [109]. Some previous studies have found that 1 g of fiber per 1000 kcal is sufficient to observe the protective actions of fiber against depression in premenopausal but not postmenopausal women [110]. Further studies are warranted to determine the quantity of fiber intake to show its preventive actions against depression.

Fruits and vegetables are critical sources of vitamins, especially for the antioxidant vitamins C (ascorbic acid) and E (Tocopherol) along with those from the B complex, with the exception of vitamin B12, which is found in animal-based products and fortified cereals or nutritional yeast [111]. Indeed, prior works have denoted that a daily intake above 400 g of fruits or vegetables ensures high blood concentrations of these vitamins [112]. Similarly, vegetables (prominently dark green leafy vegetables) are a critical source of vitamin K1 (Phylloquinone) and colorful fruits and vegetables are rich in provitamin A (beta carotenoids), a precursor of vitamin A in the body [113]. However, fruits and vegetables lack significant quantities of vitamin D, which may be obtained from animal-based foods, fungi, or some algae [114].

Vitamins are major mediators of mental health, likewise, influencing gut microbiota and SCFAs production [115]. Thus, some studies have observed potential preventive or antidepressant effects derived from vitamins. For instance, some researchers have found slight benefits from supplementation with B6 and B12 alone or integrated within a vitamin B complex [116,117]; although, other data suggest that vitamins from the B complex may provide some benefits for stress but not for depressive symptoms [118]. However, it is common that patients with MDD present low B1, B2, B3, B6, B9, and B12 vitamin levels, and, in turn, these deficits can promote the pro-inflammatory status of the immune system, participating in the pathophysiological changes related to MDD [119]. Furthermore, there is some initial evidence supporting supplementation with vitamin A and E as antidepressants, especially due to their antioxidant and anti-inflammatory effects [72], and the use of vitamin C as an adjuvant with antidepressants has obtained controversial results, with benefits in children in combination with fluoxetine, but not in adults [120].

Vitamin K has presented potential preventive actions, having been demonstrated that people with a high intake of this vitamin (>232 μg/day) had significantly lower odds of developing depressive symptoms at baseline, and at each per 100 μg/day, the odds of this condition decrease by 12% [121]. Vitamin supplementation may be interesting for individuals with some type of nutritional deficit or who have a poor nutritional status. However, due to the presence of multiple vitamins and other critical nutrients in fruits and vegetables, we think that for the general population, it is much more interesting to encourage the consumption of the recommended fruit and vegetable intake per day instead of focusing on supplementation.

The last group of nutrients reviewed in this section are polyphenols. Polyphenols are organic compounds abundantly found in plants playing critical anti-inflammatory and antioxidant properties with a promising, protective, and therapeutic role in many diseases [122]. Common polyphenols in the diet include flavonols (quercetin in onions, apples, and tea), hydroxycinnamates (coffee, many fruits) flavanols (cocoa, tea, apples, and broad beans), flavanones (hesperidin in citrus fruit), and anthocyanins (berries) [123]. The relevance of many of these polyphenols is being studied as promising agents in the field of MDD. Curcumin is one of the best-studied polyphenols in this sense, and there are some clinical trials providing evidence that 1000 mg/day of curcumin may be used as adjunctive therapy for patients with MDD; although, these benefits were not observed at lower doses (500 mg/day) [124]. However, despite there being some interesting results on the effects of curcumin as an antidepressant, further studies should be conducted to better assess this relationship, particularly in Western countries [125].

Resveratrol, a well-known polyphenol predominant in red grapes, also has cumulative evidence of antidepressant effects in preclinical models at doses between 10 and 80 mg/kg/day; although, higher doses had the most significant benefits [126]. Similarly, Wang et al. [127] used an extract from Concord grape juice, grape seed extract, and trans-resveratrol and observed that two components, dihydrocaffeic acid (DHCA) and malvidin-3′-O-glucoside (Mal-gluc), exerted antidepressant effects in mice. Interestingly, they reported how both components modulate the epigenome of their model. DHCA reduces pro-inflammatory interleukin 6 (IL-6) by inhibiting DNA methylation at the CpG-rich IL-6 sequences introns 1 and 3, whereas Mal-gluc promoted histone acetylation of the regulatory sequences of the Rac1 gene, thereby modulating synaptic plasticity.

Quercetin, a type of flavonol that has also been tested as a potential antidepressant in vivo, ameliorates lipopolysaccharide (LPS)-induced depressive rats, leading to the upregulation of BDNF, as well as other molecular markers [128].

Anthocyanins, mainly found in berries, are also being investigated in mice models as prominent antidepressants, upregulating monoamines and neurotrophic factors such as brain-derived neurotrophic factor (BDNF), which plays a critical role in MDD [129]. These are only a few examples of how polyphenols may aid in the clinical management of MDD.

More interestingly, there are more than 8000 recognized polyphenols and the function and mechanisms of many of them have not been fully elucidated [130]. Polyphenols are perhaps one of the most interesting compounds to be explored and plant-derived foods are particularly rich in these types of nutrients. Thus, an adequate dietary intervention should include fruits and vegetables to take advantage of the multiple benefits of polyphenols, vitamins, fiber, and other compounds present in these foods.

#### 2.1.3. Coffee and Tea: The Role of Caffeine and Specific Bioactive Compounds

Coffee and tea, apart from water, are the most consumed beverages worldwide, constituting the top sources of caffeine and antioxidant polyphenols in the American diet [131]. The polyphenols found in tea are named catechins, and the most important are epigallocatechin-3-gallate (EGCG), epicatechin-3-gallate (ECG), epigallocatechin (EGC), and epicatechin (EC) [132]. Other components of note in tea are theaflavins, flavonol glycosides, L-theanine, caffeine, theobromine, and volatile organic substances [133]. Among the most important components in coffee, the role of certain polyphenols should be highlighted, especially chlorogenic acids (in green beans) and caffeic acid (in roasted coffee beans), alkaloids (caffeine and trigonelline), and diterpenes (cafestol and kahweol) [134]. Due to their wide variety of beneficial components, either tea or coffee are considered critical beverages to include in the diet, and more positive effects can be reported when including both drinks [135]. Regarding daily recommendations, a prior meta-analysis found a nonlinear J-shaped relation between coffee consumption and risk of depression with a peak of protective effect for 400 mL/day [136]. Likewise, more than three cups of tea/week also seemed to exert relevant antidepressant effects [137].

Among their actions in the organism, coffee and tea seem to exert an important neuroprotective role, lowering the risk of suffering from cognitive decline and brain disorders [138,139]. Indeed, there is compelling evidence that the high consumption of coffee and tea provided reduced the risk of suffering from MDD [140]. Caffeine is one of the major determinants of these neuroprotective and antidepressant actions. In more detail, a nonlinear response between caffeine consumption and depression was found, showing their most benefits above 68 mg/day and below 509 mg/day [141]. Moreover, caffeine has also been studied as adjunctive therapy with antidepressants, showing some promising results in preclinical and clinical models [142,143]. Among the multiple mechanisms of action of caffeine, it seems that its consumption affects the epigenetic status of immune cells and also of neurons and glial cells [144,145]. It is still unknown if these epigenetic effects are related to the non-selective adenosine antagonist action of caffeine; however, this interplay appears to be promising for the treatment of motivational and dopaminergic dysfunction in depression [146]. Nevertheless, excessive consumption of coffee and caffeine has also been noted for some serious adverse effects such as anxiety, headache, increased blood pressure, nausea, and restlessness [147], as well as negatively influencing the development of the brain at early stages [148]. More studies are needed to evaluate the therapeutic safety and efficacy of caffeine in the field of MDD.

Polyphenols contained in coffee and tea can also be of great aid to the multiple benefits of these beverages. For instance, EGCG has proven some critical antidepressant effects in preclinical models, leading to an augmented BDNF, serotonin, and reduced stress hormones [124,149]. However, the major benefits observed in green tea are due to the interaction of EGCG with other catechins, teasaponin, L-theanine, and theaflavins, as they may also favorably regulate the dopaminergic system and attenuate inflammation through the downregulation of NF-κB signaling [150]. Caffeic acid also provides significant antidepressant effects in vivo, especially when combined with antidepressants [151]. Chlorogenic acid found in green coffee appears to be a major inhibitor of monoamine oxidase A (MAO-A), upregulating serotonin levels and providing antidepressant actions [152].

Future studies can be directed to describe the therapeutic and preventive mechanisms of tea and coffee consumption, with a special focus on their epigenetic mechanisms. However, the evidence appears to support that both coffee and tea and some of their components may exert antidepressant benefits. It is likely that patients with MDD may benefit from including regular doses of tea and coffee, including both drinks daily and in the morning. However, it should be equally important to consider the individual effects of coffee and tea, without the heavy drinking of these beverages. More data are required to find a proper dose of coffee and tea to prevent or support the clinical management of MDD.

#### 2.1.4. Psychobiotics: Relevance of Probiotic Food in MDD

Psychobiotics are a group of probiotics with favorable effects on the CNS function and behaviors mediated by the MGB axis, acting through immune, humoral, neural, and metabolic pathways [153]. Thus, psychobiotics can directly influence many of the pathophysiological mechanisms involved in MDD, modulating the HPA axis, inflammation, neurotransmitter production (monoamines, GABA, glutamate, and acetylcholine), BDNF levels, as well as host metabolism and systemic actions via the vagus nerve or through critical microbial metabolites such as SCFAs [154]. Psychobiotics are mainly represented by lactic acid bacteria (LAB), bacteria from the order of Lactobacillales and Bifidobacteria [155], which they can be found in supplements—using single or multispecies formulas—or included in fermented foods and beverages such as in dairy products (yogurt and kefir), soy derivates (i.e., tempeh), and other products [156]. In this section, we will explore some of the most relevant psychobiotic potential of fermented food and beverages.

On the one hand, yogurt and kefir are two groups of fermented dairy products with many benefits for human health [157]; yogurt is particularly rich in *Streptococcus thermophilus* and *Lactobacillus delbrueckii* subsp. *bulgaricus* [158]. Sometimes they can also have added LAB species such as *Bifidobacterium animalis* subsp*. lactis*, boosting their global benefits [159] Kefir is a fermented milk that comes from kefir grains, which include a specific and complex mixture of lactic acid and acetic acid bacteria that appears in symbiosis with lactose-fermenting and nonfermenting yeast [160]. Interestingly, kefir without bacteria or yeast in its composition seems to critically limit the benefits from this product [161]. Prior works have demonstrated that 3 weeks with daily consumption of probiotic yogurt improved the mood of those whose mood was initially poor [162]. Mohammadi et al. [163] observed that whereas probiotic yogurt displayed significant benefits on mental health by positively modulating the HPA axis, conventional yogurt did not have any benefit, suggesting the need of improving the probiotic formula of certain yogurts.

Likewise, a cohort of 14,539 men and women during more than 9 years from the SUN (Seguimiento Universidad de Navarra) project observed that whole-fat yogurt, but not low fat, was related to a decreased risk of depression in women, once again supporting the relevance of the entire food matrix in the benefits of a food product [164]. Moreover, the combination of yogurt plus exercise in healthy individuals led to greater increases in serotonin levels and reductions in triglyceride and high-sensitivity C-reactive protein (CRP) levels, relative to those observed for yogurt or exercise alone, demonstrating the synergic role of different lifestyle factors in the prevention of depression and inflammatory conditions [165].

Simultaneously, kefir has demonstrated a major modulatory role in the MGB axis, influencing host behavior, serotonin synthesis, immune system composition, and GABA levels linked to an increased prevalence of *Lactobacillus reuteri* [166]. Apart from their microbial composition, kefir peptides appear to be a promising antidepressant tested in vivo, activating the BDNF/phosphorylated tropomyosin receptor kinase B (TrkB) signaling pathway [167]. Further clinical studies are needed to clarify the benefits of yogurt and kefir in patients with MDD due to their promising actions.

Soy-based fermented products are mainly represented by a wide variety of traditional Asian foods such as doenjang (soybean paste or Japanese miso), ganjang (soy sauce), tempeh, or natto. Soybean contains a broad spectrum of bioactive compounds such as isoflavones, soyasaponins, lignans, cinnamic acid derivatives, terpenes, and sterols, along with antinutrients such as phytates, trypsin inhibitors, and lectins [168]. Through the fermentation process mediated by LAB, most of these antinutrients are reduced and novel compounds are generated, which, together with direct effects in the MGB axis, aid to explain the global benefits of these products [169]. Observational studies have proven that traditional Japanese dietary practices, with a high adherence to fermented soy products, are associated with lower rates of depressive symptoms [170]. In the same manner, different studies have demonstrated the efficacy of using fermented soy-based products in the clinical management of depression and cognitive impairment, with which it appears commonly associated [171]. In this line, Hwang et al. [172] conducted a 12-week intervention of 800 mg/day of a mixture of fermented soybean powder and Lactobacillus plantarum C29 (1.25 × 10^10^ CFU/g). Interestingly, they reported that the intervention group reported greater improvements in cognitive performance, being associated with higher BDNF levels. Likewise, a study using soy-based milk by Lactobacillus brevis FPA 3709 (1 × 10^6^ CFU/mL) demonstrated antidepressant efficacy in Sprague-Dawley (SD) rats [173]. This effect was achieved due to the increase in GABA levels, demonstrating similar effects to the administration of the antidepressant fluoxetine. Further studies can be directed to explore further combinations and applications of soy-derived psychobiotics in the field of MDD.

Finally, other psychobiotics have been explored to aid in the clinical management or to prevent the onset of neuropsychiatric maladies. Although less studied, the role of certain psychobiotic beverages such as water kefir or kombucha is starting to be unraveled. Water kefir is a sparkling, acidic beverage produced by fermenting a sucrose solution, to which dried fruits and water kefir grains are added. Presumably, the benefits of this beverage are similar to those reported by milk kefir, being a healthy vegan alternative, but further knowledge on the understanding of the biological dynamics of water kefir fermentation is required [174]. Regarding kombucha, this beverage is formed by the tea fermentation of *Camellia sinensis* and by a biofilm of cellulose, containing a symbiotic culture of bacteria and yeast (SCOBY) [175]. In preclinical studies, kombucha has proven antioxidant and antiaging properties, antimicrobial activity, antihypertensive, anti-inflammatory, neuroprotective, and many other benefits ([176]. In this sense, the administration of kombucha appears to mitigate the inflammatory response mediated by LPS, reducing the levels of tumor necrosis factor-α (TNF-α) and interleukins IL-1β and IL-6, restoring the levels of T cells and macrophages, and inhibiting NF-κB signaling [177]. Patients with MDD can exhibit high levels of LPS in their bloodstream (endotoxemia), associated with systemic inflammation and neuroinflammation [56,178]. The anti-inflammatory effect of kombucha should be further studied in patients with MDD, especially regarding endotoxemia and its subsequent immune response. Moreover, in a rat model, kombucha seemed to increase the integrity of the blood–brain barrier (BBB) and decrease the brain and serum levels of the malondialdehyde (MDA), a marker of oxidative stress [179]. Interestingly, both an enhanced BBB permeability and changes in MDA levels have been observed in patients with MDD [180,181]. However, despite the promising role of kombucha obtained in preclinical studies, the available clinical evidence is still insufficient and future studies should be conducted on human beings to extrapolate the benefits reported previously mentioned [182]. Another potential example of the benefits of psychobiotics is the consumption of fermented *Laminaria japonica*. In a study conducted by Reid et al. [183], subjects received 1.5 g/day of fermented Laminaria japonica for 6 weeks, displaying enhanced GABA and antioxidant activity, with neuroprotective effects.

Collectively, the role of psychobiotic foods in the clinical management and prevention of depression remains to be deeply studied. However, there are some promising results obtained in this field; preclinical and future studies should mostly be directed to unravel potential applications, doses, and recommendations.

#### 2.1.5. Dietary Supplements

Apart from food, there have been some dietary supplements explored in the context of MDD that can also work as nutraceuticals. The dietary supplements with the most evidence proven as antidepressant adjuvants are S-adenosylmethionine (SAMe), 5-methyltetrahydrofolate, ω-3 PUFA, and vitamin D [184]. Likewise, there are meta-analyses supporting the relevance of acetyl carnitine as an antidepressant in comparison to a placebo, with few adverse effects [185]. Creatine and some amino acids have also provided some clinical effects as antidepressants, in trace and ultra-trace elements; although, the level of evidence is still quite low [72]. Not only psychobiotics, but also prebiotics and postbiotics are being deeply investigated, due to the critical role of the MGB axis in MDD [62]. However, the debate about nutraceutical supplement use sometimes resides in their inconclusive evidence due to the lack of long-term studies, given the fact that their effectiveness becomes more obvious over a longer period than pharmacological drugs. In favor of bioactive components, nutraceuticals, or supplements, there is a rising research interest in them nowadays due to their having minimal side effects compared to drugs. In this sense, we encourage further studies evaluating the long-term effects of these components.

Collectively, isolated nutraceuticals present mixed evidence, and it seems that in cases of certain nutritional deficits, the benefits are greater. In this sense, we want to remark on another critical point, some studies have failed to find any significant benefit from using multi-nutrient or nutraceutical combinations in patients with MDD [186,187]. These observations could be due to the fact that despite the benefits from foods residing on particular nutrients and components of them, the concept of the food matrix is essential to understand how these compounds exert their effects in the organism. This should be taken into consideration for future nutraceuticals design, either using foods with their complete matrix or somehow emulating their matrix in designed products.

Likewise, the use of certain medicinal plants has also been explored in the field of MDD. Particularly, extracts from *Hypericum perforatum* (St John’s wort) are widely used for the treatment of depression of varying severity. Indeed, for patients with mild-to-moderate depression, St John’s wort has comparable efficacy and safety when compared to antidepressants, causing fewer adverse events [188]. However, the level of evidence on its long-term efficacy is limited, as most studies only ranged from 4 to 12 weeks. In addition, it is also unclear if this plant would be beneficial for patients with severe depression, high suicidality, or suicide risk [189]. *Hypericum perforatum* extracts have a plethora of bioactive compounds such as naphthodianthrones, phloroglucinol derivatives, flavonoids, bioflavonoids, proanthocyanidins, and chlorogenic acid; although, two molecules, hypericin and hyperforin, appear to be the most important antidepressant components [190]. In this sense, the following biological mechanisms have been previously described: (a) Inhibition of MAOs; (b) Inhibition of synaptosomal reuptake of amines; (c) Modulation of monoamine transporters; and (d) Regulatory actions on serotonin receptors [191]. All in all, *Hypericum perforatum* leads to profound changes in neurotransmitter levels, including monoamines, GABA, Glutamate, and acetylcholine [190]. However, as previously described, MDD entails a plethora of brain and systemic alterations involved in its pathogenesis, thereby explaining the limited benefits of this product in patients with severe depression. Not only *Hypericum perforatum,* but also other herbal products such as *Ginkgo biloba* leaves, ginseng, and so on can be used for alleviating depressive symptoms, acting through different biological mechanisms [192]. Future studies should be conducted on this promising line of research, considering their clinical applications in patients with MDD.

Finally, the use of dietary supplements is common in patients with MDD, especially in women; and frequently not just single, but different supplements have proven effective [193]. It is important to highlight that, unlike foods or drugs, dietary supplements and nutraceuticals do not need to be registered or approved by the US Food and Drug Administration (FDA) prior to production or sales. This could be associated with some important concerns. On the one hand, the quality and composition of the commercial finished products are poorer than the regulated ones [194]. On the other hand, although rare, this can bring severe consequences for some individuals. For instance, serotonin syndrome is a potentially life-threatening condition precipitated by the use of serotonergic drugs. These effects can be due to therapeutic medication use, interactions between medications or recreational drugs, or intentional overdose [195]. Indeed, there are some case reports in which the interaction between drugs and dietary supplements was associated with the co-occurrence of serotonin and acute compartment syndrome, demonstrating the remarkable issue of studying these interactions [196]. In addition, some deleterious interactions between nutraceuticals/dietary supplements with drugs in the field of MDD have been previously defined [197]. In this sense, it is critical to give more effort to the prevention of adverse outcomes derived from the use of dietary supplements and their interactions. In the last years, some countries have started detecting, monitoring, or reporting adverse events derived from dietary supplements in an activity designed as Nutrivigilance. Clinicians should be conscious about the use of dietary supplements by patients with MDD and obtain enough information before recommending some nutraceuticals/dietary supplements, taking into consideration possible or reported interactions. Overall, Nutrivigilance is a novel field that remains to be deeply explored and some caution may be required. In this line, we recommend focusing on ensuring the intake of beneficial foods and nutrients rather than supplements (if no severe deficits are reported), in order to maximize the benefits of these components

### 2.2. Dietary Strategies to Implement in Patients with MDD

Not only specific nutrients and foods, but also the entire dietary context are important approaches to consider in the clinical management and prevention of MDD. Indeed, unhealthy dietary patterns clearly influence the development and aggravation of MDD. For instance, it is well-known that excessive ultra-processed food consumption in westernized and proinflammatory diets is associated with pro-depressant effects [198,199]. On the other hand, different dietary approaches have been emerging since the last century in order to improve depressive symptoms. Some of the best-known strategies studied to support the prevention and treatment of MDD include the Mediterranean diet (MDiet), low-carbohydrate diet (LCD), and plant-based diet (vegetarianism and veganism). Here, it must be understood that the type of dietary strategy is not the most important point to consider, but the adherence to a healthy dietary pattern and the inclusion of a wide variety of food and nutrients is what really matters.

The MDiet is a type of dietary pattern mainly characterized by a richness of highly complex carbohydrates in fiber (cereals, legumes, vegetables, and fruits), polyunsaturated fatty acids with antiatherogenic and anti-inflammatory properties (i.e., fish, extra virgin olive oil, and nuts), and a plethora of bioactive compounds with antioxidative properties such as flavonoids, phytosterols, terpenes, and polyphenols [35]. This strategy has been widely explored in the prevention and management of non-communicable diseases (NCDs), especially those associated with aging [200] In this sense, some authors have described the relevance of the MDiet and some of their most representative elements on a differential epigenetic profile, which may explain the benefits of this diet in different disorders [201]. MDD is an intricate and multifactorial disorder and different results have been obtained regarding the inclusion of the MDiet. Some of the most important studies regarding the role of the MDiet in the prevention of different NCDs are PREDIMED (Prevención con Dieta Mediterránea) trials. One study described that moderate fish and long-chain ꞷ-3 PUFA consumption integrated in an MDiet was associated with lower odds of depression in a U-shaped way [202]. Likewise, one systematic review and meta-analysis found that adherence to the MDiet was prominently related to a reduced incidence of depression [203]. More detailly, another meta-analysis also found benefits from high and moderate MDiet adherence with a reduced risk of depression. Nevertheless, the positive association of moderate adherence seemed to fade with age [204]. Despite these conclusions, there are also other systematic reviews and meta-analyses that have failed to find significant associations between high adherence to the MDiet and the risk of depression [203,205], suggesting promising but still inconsistent evidence of the benefits of the MDiet in the prevention of depression. In the context of clinical management of patients with MDD, the SMILES (Supporting the Modification of lifestyle in Lowered Emotional States) trial should be highlighted. A total of 67 individuals were studied, 33 with a modified MDiet intervention and 34 without any nutritional but social support. Of them, 55 were also receiving some type of therapy, pharmacology, psychotherapy, or a combination of both. A total of 31 of the dietary support group and 25 of the controls completed the study at 12 weeks and after further analysis, the study concluded that this type of nutritional intervention helped in the clinical management of MDD, measured with the Montgomery–Åsberg Depression Rating Scale (MADRS) [206]. The dietary protocol for these patients is described in [207]. Extra virgin olive oil (EVOO), one of the most representative elements of the MDiet has recently proven antidepressant effects in patients with severe MDD, but not in those with mild to moderate [208]. All in all, the growing number of studies focusing on the preventive and therapeutic support of the MDiet has opened an interesting field of research. Further efforts are warranted to unravel the mechanisms and optimum protocols to obtain the greatest benefits from this type of dietary intervention.

LCDs such as the ketogenic or Atkins diet are mainly characterized by a reduced carbohydrate and high fat intake. Both aim to induce nutritional ketosis, and previous studies have found some evidence from the use of these strategies in the prevention and treatment of some neurological disorders such as epilepsy or Alzheimer’s disease [209]. Ketone bodies are not only a fat-derived energy supply, but they also exert important epigenetic functions, especially through histone post-translational modifications [210]. To date, there is some evidence of the antidepressant actions of ketone bodies in preclinical studies, case reports, and case series, but no clinical trials have been conducted in this field [211]. The benefits seem to be related to their regulatory role on different neurotransmitter levels, mitigating oxidative stress, insulin dysfunction, and inflammation, while favoring mitochondrial function and neurotrophic factors [211]. A negative point of following an LCD, however, may be related to negative long-term effects, as some authors have hypothesized that LCDs can induce metabolic depression, linked to reduced glycogen stores, and increased feelings of fatigue [212]. Likewise, combining this kind of strategy with intermittent fasting can exert synergic effects in the amelioration or prevention of depressive symptoms, sharing some similarities with an evolutionary or Paleolithic lifestyle; although, there are no clinical studies conducted with intermittent fasting in psychiatric populations yet [213,214]. Further efforts are required in this area before establishing the adequacy of this dietary context in MDD.

Plant-based diets also have different dietary patterns that have gained popularity in the last years, mainly aiming to reduce the environmental footprint of the diet and promote human health and animal welfare [215]. In general terms, plant-based diets are prominently represented by non-animal products; although, there is a broad spectrum of possibilities: including low meat but all food items (omnivore), all except meat (pesco-vegetarian), all except meat and fish (ovo-lacto-vegetarian), or plant-based items (vegan). Despite some evidence arising regarding the actions of a plant-based diet in the brain, the benefits and underlying mechanisms remain to be explored [216]. In this line, a possible epigenetic role of different bioactive components found in plants, especially by modulating DNA methylation, has been described [217]. There is some evidence that vegetarians/vegans may be at a higher risk of suffering from depression [218,219]. It is inconclusive if these associations can be mediated by a proper diet or if the vegans/vegetarians’ dietary pattern is a result of an increased susceptibility to depression. A potential explanation for this fact is that the vegetarian/vegan diet may not be properly balanced, being associated with the lack of some critical dietary components [220]. However, some authors argue that when well-designed and supplemented with vitamin B12, plant-based diets could bring potential benefits for overall health [221]. Once again, the quality of the diet may be a determinant factor. A cross-sectional study provided evidence that high-quality plant-based products (avoiding ultra-processed products and ensuring an adequate intake of different nutrients) can be protective for vegans/vegetarians without depression; although, individuals affected by depression did not show that benefit [222]. Similarly, avoiding the consumption of animal-based products such as meat, eggs, or dairy products did not show an improvement in mental health or MDD, according to some studies and meta-analyses [223,224]. This is attributed to the fact that these natural foods are rich in the different above-mentioned nutrients with potential benefits for the body [225]; although, vegetarianism/veganism if properly balanced, is also a laudable choice. Overall, it must be added that the actual studies in this area are quite heterogeneous. Currently, for patients with MDD, it seems that limiting or avoiding animal products may not be the appropriate approach. However, further research is needed, especially on well-designed and balanced vegetarian/vegan diets, before any final conclusions can be drawn.

Collectively, different studies regarding the implementation of a dietary pattern to prevent and ameliorate depression have been conducted. The MDiet has been the most widely studied strategy, providing many promising results; although, the level of evidence of its benefits is still insufficient. There are few studies on LCDs in depression; although, they may have some short-term benefits from depression due to the multiple regulatory benefits of ketone bodies. Finally, it seems that there is a negative association between a plant-based diet and depression; however, there is great heterogeneity in the current literature. Moreover, it is not well-understood if vegetarianism/veganism can be a consequence of, or contributes to, some pathophysiological mechanisms. The most important points to consider are both the adherence to a healthy dietary pattern and, more prominently, the inclusion of high-quality foods, avoiding ultra-processed products, and ensuring a complete nutritional content, which will bring the most benefits for the prevention and, in an adequate context, for the clinical management of MDD.

## 3. Conclusions

**Figure 2:**
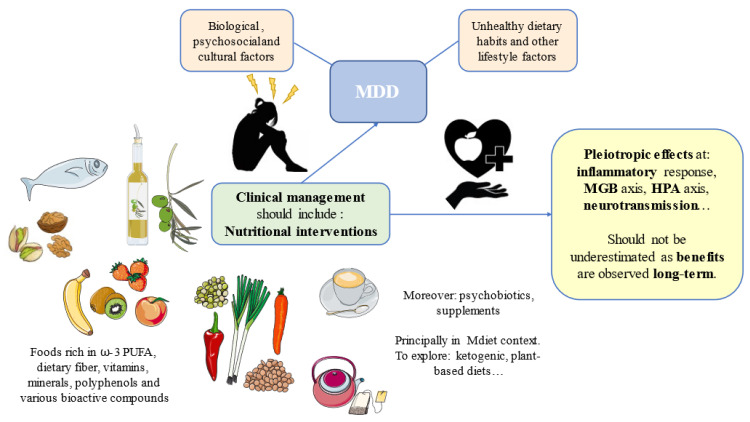
Summary of the main components of promising nutritional interventions and their pleiotropic effects in the pathophysiology of depression. MDD: Major Depressive Disorder; HPA: hypothalamic–pituitary–adrenal; MGB: microbiota–gut–brain; ꞷ-3 PUFA: omega 3 polyunsaturated fatty acids.

MDD is a complex neuropsychiatric disorder with many biological, psychosocial, and cultural factors implicated. A mismatch of our evolutive design—for instance, following unhealthy dietary patterns with a low presence of certain critical nutrients—can contribute to the onset and development of this complex disorder. In this context, dietary interventions can potentially aid to prevent and improve the clinical management of patients with MDD, as summarized in Table 1. There are foods of special interest such as fish, nuts, seeds, EVOO, fruits, vegetables, coffee, and tea, which have been widely explored in the field of MDD. These foods are especially rich in critical nutrients such as ꞷ-3 PUFA, dietary fiber, vitamins, minerals, polyphenols, and various bioactive compounds with direct and indirect implications on the different pathophysiological mechanisms of MDD. For instance, by restoring neurotransmitter levels, targeting the HPA axis, attenuating the inflammatory response, or modulating the MGB axis, among others. Most of these foods and nutrients are encompassed under the MDiet pattern, and growing evidence supports the protective and adjuvant role of this diet in many NCDs, including in certain neuropsychiatric maladies. Other groups of foods or dietary strategies, such as psychobiotics found in fermented products, certain dietary supplements, or ketogenic and plant-based diets, are being currently investigated in the field of MDD. However, it should be noticed that nutrition is a complex research area in which most studies are preclinical or observational, and the greatest benefits from this lifestyle habit are observed over the long term (Figure 2). As the great physician, Hippocrates said, “Illnesses do not come upon us out of the blue. They are developed from small daily sins against Nature. When enough sins have accumulated, illnesses will suddenly appear.” Nevertheless, due to the promising role of diet in the prevention and management of MDD, we encourage further studies in this growing area in combination with other lifestyle medicine approaches, aiding to prevent and revert these daily “sins”.

## Figures and Tables

**Table 1:** **Main nutrients and dietary recommendations** with potential antidepressant effects.

Nutrients	Food	Intake Recommendations (Food)	Antidepressant Mechanisms	Studies in MDD	References
**Omega 3 PUFA**	Fish and seafood (DHA and EPA), seeds and nuts (ALA)	Two servings of fish per week or 350 g weekly including in which 200 g may be from oily fishes. 30 g/day of mixed nuts (15 g walnuts, 7.5 g hazelnuts, and 7.5 g almonds) comprised in a healthy dietary pattern appears to be an important recommendation to prevent MDD.	Epigenetic modulation; Anti-inflammatory; Prebiotic; Increase in membrane fluidity; serotonin transport; enhanced dopamine concentration and dopamine 2 receptor in the frontal cortex; cellular signaling.	**DHA and EPA** either by supplementation or contained in a high intake of fish (≥2 times per week exerted relevant protective and antidepressant effects. **ALA** contained in seeds and nuts (especially walnuts) can ameliorate depressive symptoms and prevent their onset.	[78,79,80,81,82,83,84,85,86,87,88,89,90]
**Vitamin D**	Fish and animal products; mushrooms; fortified products	Two servings of fish per week or 350 g weekly including in which 200 g may be from oily fishes.	Vitamin D influences the immune and gut microbiota modulation; Serotonin synthesis; Circadian clock regulation and augmented BDNF production, exerting synergic effects with omega 3 PUFA.	Controversial results have been obtained regarding the possible antidepressant effect of **vitamin D** intake; although, its low consumption worldwide can be involved in the high prevalence of MDD.	[91,92,93,94,95,96,97,98,99]
**Vitamins from the B complex**	Fruits and vegetables/Animal products or supplementation (B12)	800 g per day of fruits and vegetables are recommended as part of a healthy dietary pattern.	Anti-inflammatory and pleiotropic actions.	Low levels of **B1**, **B2**, **B3**, **B6**, **B9**, and **B12** vitamins can be related to a proinflammatory status of the immune system. Some studies have found slight benefits from supplementing with B6 or B12 alone or integrated into the B complex. However, daily intake above 400 g of fruits or vegetables is the most adequate approach to elevate the serum levels of vitamins	[114,115,116,117,118]
**Other vitamins** **(A, C, E, K)**	Fruits and vegetables, animal products	800 g per day of fruits and vegetables are recommended as part of a healthy dietary pattern.	Antioxidant, anti-inflammatory, and pleiotropic effects	There is some preclinical evidence supporting the supplementation with **provitamin A and E** as antidepressants; **Vitamin C** has been explored as an adjuvant with fluoxetine in children but not in adults; High intake of **vitamin K** (>232 μg/day) had significantly lower odds of developing depressive symptoms at baseline and each per 100 μg/day the odds of this condition decreased by12%. Daily intake above 400 g of fruits or vegetables is the most adequate approach to elevate the serum levels of vitamins.	[114,118,119,120]
**Dietary fiber**	Fruits and vegetables	800 g per day of fruits and vegetables are recommended as part of a healthy dietary pattern.	Prebiotic and modulatory effects in the MGB axis; SCFA modulation.	1 g of fiber per 1000 kcal exerts protective actions of **fiber** against depression in premenopausal, but not postmenopausal women.	[108,109]
**Polyphenols**	Fruits and vegetables/Coffee and tea	800 g per day of fruits and vegetables are recommended as part of a healthy dietary pattern. A nonlinear J shape curve with a peak on 400 mL of coffee/day and more than 3 cups of tea per week.	Pleiotropic effects	1000 mg/day of **curcumin** but not 500 mg/day may be used as adjunctive therapy for patients with MDD Preclinical models have shown that doses between 10 and 80 mg/kg/day, of **resveratrol**, provide antidepressant effects; although, higher doses had the most significant benefits. **Quercetin** has provided a potential antidepressant role in vivo, ameliorating lipopolysaccharide (LPS)-induced depressive rats, leading to the upregulation of BDNF and other molecular markers. **Anthocyanins**, mainly found in berries, are being investigated in mice models as prominent antidepressants, upregulating monoamines and neurotrophic factors such as BDNF. **EGCG** has proven to have some critical antidepressant effects in preclinical models, leading to an augmented BDNF, serotonin, and reduced stress hormones. **Caffeic acid** also provides significant antidepressant effects in vivo, especially when combined with antidepressants. **Chlorogenic acid** found in green coffee appears to be a major inhibitor of the monoamine oxidase A (MAO-A), upregulating serotonin levels and providing antidepressant actions.	[121,122,123,124,125,126,127,128,129]
**Caffeine**	Coffee and tea	A nonlinear J shape curve with a peak on 400 mL of coffee/day and more than 3 cups of tea per week.	Neuroprotective; Epigenetic modulation of neurons, immune and glial cells; Targeting of the dopaminergic system through the non-selective adenosine antagonist action.	A nonlinear response between **caffeine** consumption and depression was found, showing their most benefits above 68 mg/day and below 509 mg/day.	[137,138,139,140,141,142,143,144,145,146,147]
**Psychobiotics**	Fermented foods and beverages (Yogurt, kefir, soy-derived products, kombucha, *Laminaria japonica*, etc.*)*	Fermented foods can be included in a healthy dietary pattern daily.	Modulatory effects on HPA and MGB axis; inflammation; neurotransmitter production (monoamines, GABA, glutamate, acetylcholine); BDNF levels; host metabolism.	3 weeks with daily consumption of **probiotic yogurt** improved the mood in people with poor mood and other significant benefits on mental health by positively modulating the HPA axis; Whole-fat yogurt, but not low fat, was related to a decreased risk of depression in women. **Kefir** and kefir peptides appear to be a promising antidepressant tested in vivo, displaying a major modulatory role in the MGB axis, influencing host behavior, serotonin synthesis, immune system, BDNF/TrkB signaling, and GABA levels High adherence to **soy-based fermented products** in traditional Japanese regimes is associated with lower rates of depressive symptoms, aiding in the clinical management of depression and cognitive impairment; Soy-based milk with Lactobacillus brevis FPA 3709 (1 × 10^6^ CFU/mL) demonstrated antidepressant efficacy in SD rats similar to fluoxetine. **Kombucha** can attenuate LPS-mediated neuroinflammation and oxidative stress markers, but direct evidence of its antidepressant role is needed.	[152,153,154,155,156,157,158,159,160,161,162,163,164,165,166,167,168,169,170,171,172,173,174,175,176,177,178,179,180,181]

## Data Availability

Not applicable.

## Abbreviations

ACTH	adrenocorticotropin hormone
ALA	α-linolenic acid
AST	aspartate aminotransferase
BBB	blood–brain barrier
BDNF	brain-derived neurotrophic factor
BUN	blood urea nitrogen
CCK	cholecystokinin
CNS	central nervous system
COVID-19	coronavirus disease 2019
CRF	cortisol release factor
CRP	C-reactive protein
DHA	docosahexaenoic acid
DHCA	dihydrocaffeic acid
DSM-5	Diagnostic and Statistical Manual of Mental Disorders, 5th Edition
EC	epicatechin
ECG	epigallocatechin
EGCG	epigallocatechin-3-gallate
ELS	Early Life Stress
EPA	eicosapentaenoic acid
EVOO	extra virgin olive oil
GABA	gamma-aminobutyric acid
GAL	galanin
GDNF	glial-derived neurotrophic factor
GHDx	Global Health Data Exchange
HAM-D	Hamilton Depression Rating Scale
HDL-C	high-density lipoprotein cholesterol
HPA axis	hypothalamus–pituitary–adrenal
IL-1β	interleukin 1β
IL-6	interleukin 6
LAB	lactic acid bacteria
LCD	low-carbohydrate diet
LPS	lipopolysaccharide
MADRS	Montgomery-Åsberg Depression Rating Scale
Mal-gluc	malvidin-3′-O-glucoside
MAO-A	monoamine oxidase
MCH	melanin-concentrating hormone
MDA	malondialdehyde
MDD	Major Depressive Disorder
MDiet	Mediterranean Diet
MGB axis	microbiota–gut–brain axis
MNA	Mini Nutritional Assessment
NCDs	non-communicable diseases
NPS	neuropeptide S
NPY	neuropeptide Y
OXT	oxytocin
PREDIMED trials	Prevención con Dieta Mediterránea trials
SAMe	S-adenosylmethionine
SCFAs	short-chain fatty acids
SCOBY	symbiotic culture of bacteria and yeast
SD	Sprague-Dawley
SMILES trial	Supporting the Modification of lifestyle in Lowered Emotional States trial
SUN project	Seguimiento Universidad de Navarra project
TNF-α	tumor necrosis factor-α
TrkB	tropomyosin receptor kinase B
VDBP	vitamin D-binding protein
VP	vasopressin
WHO	World Health Organization
ω-3 PUFA	omega 3 polyunsaturated fatty acids
